# Principles for Optimal Electrode Design and Operation for Desalination with Electrochemical Ion Pumping

**DOI:** 10.1021/acs.est.5c15799

**Published:** 2026-02-23

**Authors:** Weifan Liu, Longqian Xu, Shihong Lin

**Affiliations:** † Department of Civil and Environmental Engineering, 5718 Vanderbilt University, Nashville, Tennessee 37235-1831, United States; ‡ Department of Chemical and Biomolecular Engineering, 5718 Vanderbilt University, Nashville, Tennessee 37235-1831, United States; § Department of Civil and Environmental Engineering, 3990 Rice University, Houston, Texas 77005, United States

**Keywords:** electrochemical desalination, electrochemical ion pumping, electrode design

## Abstract

Electrochemical ion pumping (EIP) is an emerging electrochemical separation form that eliminates solution mixing in conventional capacitive deionization and enables pseudocontinuous desalination with unidirectional ion flux. In this work, we systematically investigate electrode design and operational parameters governing the performance of EIP desalination using both experiments and modeling. We first evaluated the effect of carbon and polymer fractions on ionic and electronic transport within the cation-shuttling electrodes, demonstrating that optimal performance requires balancing ion mobility through the polymer phase and the electronic conductivity and ion storage capacity provided by the carbon phase. We then assessed the impact of electrode capacity and conductivity on EIP performance by varying the ratio of activated carbon and carbon black, showing that desalination performance is relatively stable over a wide range of capacity and is only undermined at extremely low capacity. Finally, we analyze the role of cycle time and show that EIP has similar specific energy consumption with very different cycle times as long as electrolysis is prevented. Overall, these results establish guiding principles for rational electrode design and operation to achieve optimal EIP performance.

## Graphical abstract

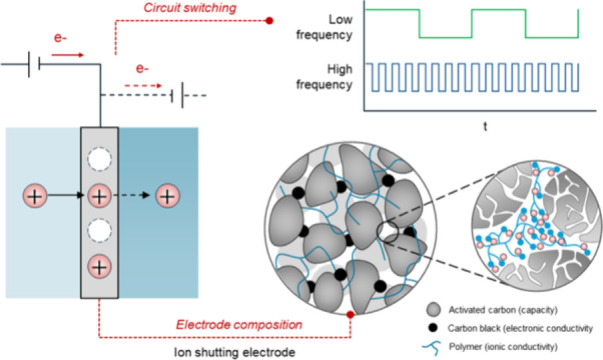

## Introduction

Electrochemical separation is an emerging research frontier with broad applications in desalination,^1–3^ contaminant removal,^4,5^ mineral extraction,^6–8^ and nutrient recovery.^9–12^ Among various electrochemical separation processes, capacitive deionization (CDI) and electrodialysis (ED) have attracted significant attention in recent years. In conventional CDI, an electric field is applied between two porous electrodes, driving ion adsorption onto the electrodes, and thereby removing ions from the feed solution. Once the electrodes are saturated, the polarity is reversed to release the adsorbed ions into a receiving stream.^1,2^ However, due to the limited ion storage capacity of the electrodes, multiple charging–discharge cycles are typically required to achieve a desirable separation.^13^ Compared to conventional adsorption or ion exchange processes, the bed volume treated in each cycle (i.e., between two regeneration steps) is very small in most CDI applications. The need to frequently switch the flow between the feed and receiving solutions leads to solution mixing, which undermines separation performance and system efficiency.^14,15^ ED avoids solution switching by driving continuous, unidirectional ion transport across ion-exchange membranes using Faradaic reactions at terminal electrodes, and has demonstrated high efficiency with large ED stacks.^16,17^ However, the reliance on terminal electrode reactions introduces additional considerations, such as gas evolution and pH management, particularly in small-scale and modular implementations.^18,19^

Electrochemical ion pumping (EIP) has been recently developed as a novel electrochemical desalination process based on capacitive ion uptake mechanism but without the need for solution switching.^20,21^ Unlike conventional CDI, which relies on physical solution switching, EIP employs circuit switching to alternate between the adsorption (charging) and desorption (discharge) steps. EIP completely avoids solution mixing and enhances desalination performance compared to CDI.^20^ Moreover, a flow-synchronized ring-shaped EIP architecture (FS-R-EIP) can eliminate terminal electrodes entirely by arranging ion-shuttling electrodes in a closed-loop configuration and synchronizing circuit switching with flow direction. This design enables effective desalination using a single power source and achieves high current and energy efficiency under small-scale operating conditions.^22^

In its simplest form, a symmetric plate-and-frame EIP cell consists of two terminal electrodes, a cation-shuttling electrode (CSE), and two anion-exchange membranes (AEMs) (Figure 1a). During the charging step, the left circuit is connected, the right circuit is disconnected, and the CSE acts as a cathode to capture cations from the diluate. To initiate the discharge step, the polarity of the CSE is reversed by circuit switching to disconnect the left circuit and connect the right circuit. In the discharge step, the CSE functions as an anode, releasing ions into the concentrate. By repeatedly switching the circuits, ions are continuously pumped from the diluate to the concentrate.^20^

**Figure 1:**
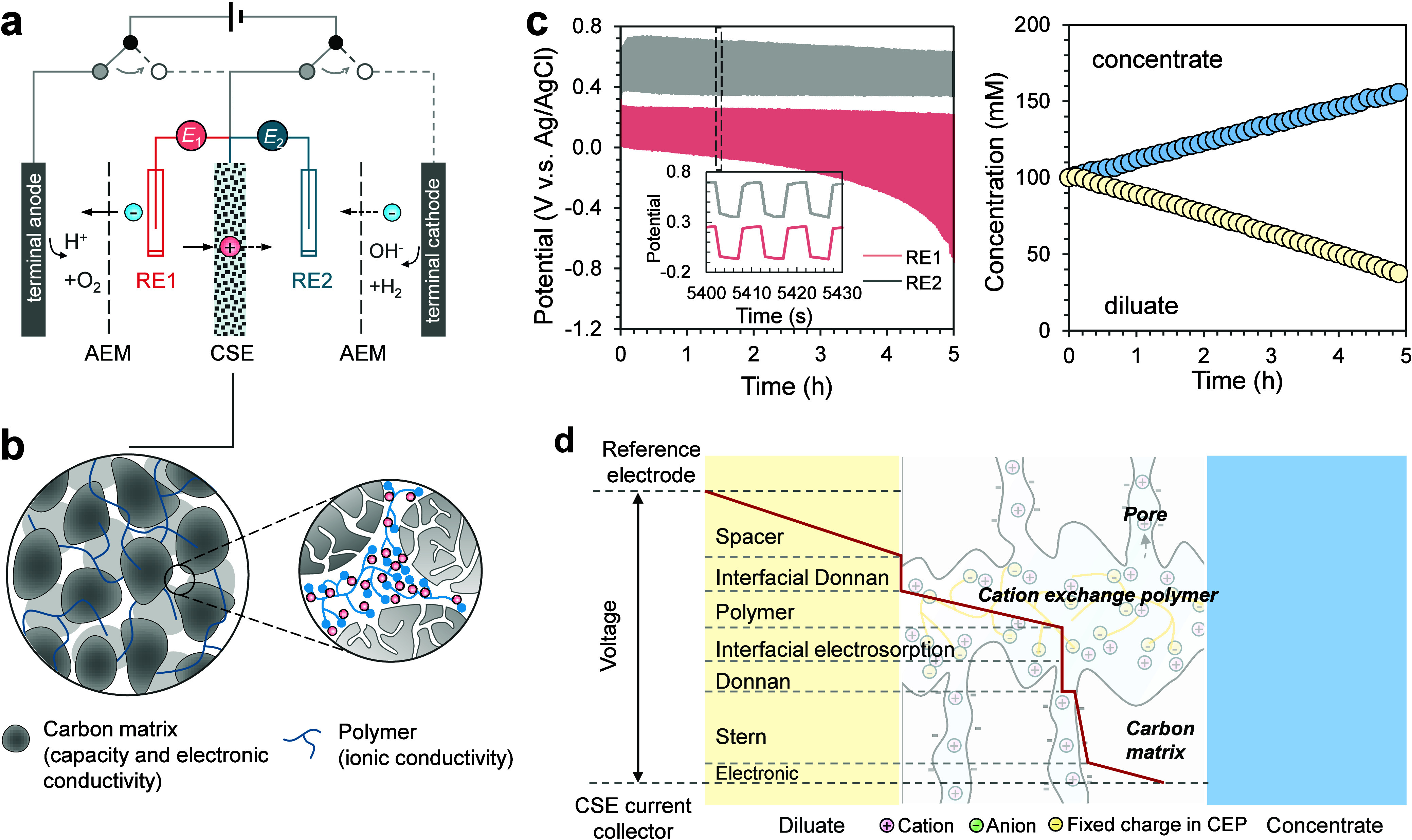
(a) Structure of an electrochemical ion pumping (EIP) cell consisting of one cation-shuttling electrode (CSE), two anion-exchange membranes (AEMs), and terminal anode/cathode. Relay-controlled circuit switching alternately charges and discharges the electrodes, driving directional ion transport between diluate and concentrate streams. Two reference electrodes (RE1 and RE2) were included only for monitoring the CSE potential during experiments and are not required for practical EIP operation. (b) Schematic of the composite electrode structure, where ion-exchange polymer (blue) serves as ion transport pathways, while activated carbon particles provide electronic conductivity and ion storage capacity. (c) Representative electrode potential profiles versus Ag/AgCl reference electrodes (left) and corresponding evolution of diluate and concentrate concentrations (right) over time. The red and gray traces denote the CSE potential versus two Ag/AgCl reference electrodes (RE1 and RE2) in the diluate channel and concentrate channel, corresponding to the two circuit connection states. The gradual increase in potential magnitude in diluate channel reflects the increasing ionic resistance during sustained desalination under constant-current operation. The inset highlights the rapid and periodic switching of electrode potentials achieved by electronic relays. The current density is 40 A m^–2^. (d) Qualitative potential distribution from the reference electrode to the current collector in a CSE of an EIP system. The red line indicates the potential profile across different interfacial regions, including the spacer channel, cation-exchange polymer, and porous carbon matrix. In this example, the electrode adsorbs cations from the diluate stream.

Although EIP shares the same fundamental ion storage mechanism as conventional CDI, it features distinct electrode design and operational characteristics.^20,21^ CDI electrodes are typically composed of porous carbon or intercalation materials, with carbon black added to enhance electrical conductivity.^23,24^ Polyvinylidene fluoride is often used as a binder for mechanical integrity.^25^ In contrast, the CSE in EIP is made of carbon particles with its macropores (i.e., voids between carbon particles) fully filled with an ion-exchange polymer (IEP) (Figure 1b).^20,21^ In such composite electrodes, IEP provides ion transport pathways while sealing macropores to prevent cross-permeation between diluate and concentrate, whereas carbon particles provide the electronic conductivity and ion storage capacity. While the basic principles regarding what properties a CSE entails have been established in a previous proof-of-concept paper,^20^ the influence of specific electrode composition and operational conditions (e.g., switching frequency, current density) on EIP performance remains largely unexplored.

The carbon fraction is a critical design parameter. A higher polymer fraction (i.e., lower carbon fraction) facilitates ion transport but undermines the percolation network for electron transfer and the capacity for ion storage. In contrast, a higher carbon fraction improves ion storage capacity and electron conductivity but may hinder ion transport. Identifying the optimal balance between polymer and carbon fractions is therefore essential for maximizing EIP performance. Similarly, the choice of carbon material is important: activated carbon (AC) generally provides higher ion storage capacity, whereas carbon black (CB) offers superior electrical conductivity.^26–29^ While electrode capacity is a critical performance parameter in CDI,^30^ its role in EIP is less clear: because EIP can operate with very short cycle time with fast circuit switching, capacity appears to be less important than in CDI which prefers to operate with a long adsorption half cycle to minimize solution switching and mixing. Therefore, we postulate that EIP can achieve efficient desalination even with relatively low-capacity electrodes. As a unique feature of EIP, fast circuit switching and short cycle time may also influence energy consumption and mitigate the risk of electrolysis by limiting voltage rise due to reduced electrode saturation.

To elucidate the factors governing electrode design for high-performance EIP, we conduct a systematic study combining experiments with mathematical modeling. We first investigate the effects of carbon fraction on ion transport and electrode resistance, then examine the role of electrode capacity by varying the ratio between activated carbon and carbon black, and finally evaluate the influence of switching frequency on energy consumption. Here, desalination is used as a model system for its simplicity to reveal the governing electrode-level trade-offs intrinsic to the ion-shuttling mechanism of EIP. The insights obtained are intended to establish general design and operational principles for ion-shuttling electrodes, which are applicable across different EIP architectures and relevant to a broader range of electrochemical separation applications beyond desalination.

## Materials and Methods

### Materials

LIQUion dispersion (LQ-1115-1100 EW, 15% Nafion in a mixture of water and ethanol) was obtained from Ion Power, Inc., USA. AC powder with particle size of 5 ± 1 μm and surface area of 1800 ± 100 m^2^ g^–1^ was purchased from XFNANO, Ltd. (USA). CB powder was purchased from Canrud (China) and has a BET surface area of 62 m^2^ g^–1^, a powder conductivity of approximately 10–15 S cm^–1^, and a primary particle size of 40–50 nm. Titanium mesh (100 mesh size, thickness 250 μm), purchased from Jiangxin Wire Mesh Products Co., Ltd. (China), served as the current collector. Graphite plates, supplied by Beijing Jinglong Special Carbon Technology, were used as terminal electrodes. AEMs (AM­(H)-PP membranes RALEX), with a thickness of 400 μm, were purchased from Membrain (Czech Republic).

### Fabrication of Cation-Shuttling Electrode

To investigate the influence of electrode properties on EIP desalination performance, we fabricated polymer-carbon composite electrodes with varying compositions. First, we used either AC or CB as the carbon phase and systematically varied the carbon weight fractions in the polymer-carbon mixture. In a separate set of electrodes, we varied the ratio of AC to CB in the carbon phase while keeping the overall carbon fraction (i.e., weight of carbon vs the total weight of carbon and polymer) constant. The carbon powders were ground in a quartz mortar to reduce agglomeration and promote uniform dispersion. Then they were mixed with a Nafion solution to form a uniform slurry, which was then applied to a 100-mesh titanium mesh substrate (6 cm × 6 cm) with a consistent thickness. The coated electrodes were air-dried for 12 h and subsequently hot-pressed at 140 °C under 27 MPa for 30 min. The resulting electrodes had a thickness of 350 ± 40 μm. The total mass loading of the electrode, including both carbon and polymer, was approximately ∼40 ± 2 mg cm^–2^. Waterproof tape (3M, USA) was used to frame the electrodes, with an effective surface area of ∼6 cm^2^ exposed on both sides. Prior to electrochemical testing, the electrodes were stored in DI water.

### EIP Desalination Experiments

The single-electrode EIP cell consists of a pair of graphite plate terminal electrodes, two AEMs, one CSE, and four spacers (detailed configurations shown in Figure S1). Two electrical relays were used to control the circuit switching. Experiments were performed in semibatch mode, i.e., a solution flowing through a channel in the EIP cell is sent back to the reservoir. The open flow channels between the AEMs and the CSE, referred to as spacer channels in this study, are defined by PTFE channel frames with a thickness of 10 mm. These frames include two side openings for mounting Ag/AgCl reference electrodes. No porous spacer material was inserted in the channels. The diluate (30 mL) circulated between the diluate tank and the diluate channels of the EIP cell, while the concentrate (30 mL) circulated between the concentrate tank and the concentrate channels of the EIP cell. The electrode rinse solution (200 mL 100 mM NaCl) circulated between the terminal anode and cathode channels as well as the external electrolyte tank. The initial salt concentration of the feed solution in the diluate and concentrate channels/tanks was 100 mM. The flow rates of both the diluate and concentrate streams were 1 mL s^–1^. Conductivity, current, and electrode potential were monitored with the Sensor Kit (Vernier Software & Technology, USA), which includes data collectors, conductivity sensors, current sensors, and potential sensors. The salt concentrations were calculated based on a calibration curve relating conductivity to salt concentration. Representative electrode potential profiles versus Ag/AgCl reference electrodes and corresponding evolution of diluate and concentrate concentrations over time are shown in Figure 1c. The average voltage ( $\overset{-}{V}$ ) over many charging and discharge cycles is defined as

$$\overset{-}{V}=\frac{\sum \int_{0}^{t_{\mathrm{hc}}}V_{\mathrm{ch}}(t)dt+\sum \int_{t_{\mathrm{hc}}}^{t_{c}}V_{\mathrm{dis}}(t)dt}{\Delta t}$$ 1

where *V*_ch_(*t*) is the potential drop between the reference electrode and CSE for charging and *V*_dis_(*t*) is the potential drop between the CSE and reference electrode for discharge at current density *i*, *t*_hc_ is the half-cycle time which is equal for charging and discharge and *t*_c_ is the full-cycle time comprising one charging and one discharging half-cycle.

The performance of a CSE is evaluated by the desalination rate (ion flux) and energy efficiency (specific energy consumption), based on approximately 20% salt removal to enable quick and consistent comparison. Specifically, ion flux (*J*_i_, mol m^–2^ s^–1^) is defined as trans-CSE salt flow rate normalized by the CSE area:

$$J_{i}=\frac{\left(c_{f}-c_{d}\right)v_{d}}{A\Delta t}$$ 2

where *c*_f_ and *c*_d_ are the molar salt concentrations in the feed and diluate streams, respectively, *v*_d_ is the volume of the diluate, *A* is the effective electrode area and Δ*t* is the total duration (Δ*t* = ∑∫_0_^*t*_hc_^ d*t* + ∑∫_*t*_hc__^*t*_c_^ d*t*). Current efficiency (η), defined as the percentage of electrical current attributable to ion flux, is calculated as

$$\eta =\frac{i}{2FJ_{i}}$$ 3

where *i* is the applied current density (A m^–2^) and *F* is the Faraday constant (∼96,485 A s mol^–1^). The factor of 2 accounts for the half-cycle operation of EIP: due to circuit switching, only one-half of the system is electrically active at any given time and contributes to either ion adsorption or desorption. To correctly relate the time-averaged applied current to the measured ion flux (desalination rate) over many charging-discharge full cycles, a factor of 2 is included.

The energy efficiency of EIP process can be quantified using ion-specific energy consumption, (SEC_*i*_), defined as the energy consumed to remove a unit mole of NaCl:

$${SEC}_{i}=\frac{i\left(\sum \int_{0}^{t_{\mathrm{hc}}}V_{\mathrm{ch}}(t)dt+\sum \int_{t_{\mathrm{hc}}}^{t_{c}}V_{\mathrm{dis}}(t)dt\right)}{J_{i}\Delta t}$$ 4

### Electrode Characterization

Cyclic voltammetry (CV) was performed on the composite CSE (area ≈ 1 cm^2^, mass ≈ 3 mg) in 1 M NaCl electrolyte to estimate intrinsic electrode charge storage capacity, using an Ag/AgCl reference electrode and a platinum (Pt) counter electrode. The high electrolyte concentration was chosen to minimize solution resistance and transport-induced potential distortion during CV measurements, allowing reliable estimation of intrinsic electrode charge storage. While double-layer charge storage may exhibit some dependence on ionic strength, the CV-derived capacity here is used as an intrinsic or upper-bound material parameter, whereas the effective charge utilization during desalination is evaluated directly under 0.1 M NaCl conditions through coupled transport and operational constraints. The potential was swept between 0.2 and 1.0 V at 10 mV s^–1^ for five consecutive cycles using a Bio-Logic SP-150 potentiostat, and the stabilized CV curve was used to calculate the electrode capacitance. Electrochemical impedance spectroscopy (EIS) was carried out on a new composite CSE under the same conditions, using a 10 mV sinusoidal perturbation at 0.2 V vs open circuit potential over the frequency range of 100 kHz to 10 mHz.

## Theory

A dynamic ion transport model for the EIP process was developed in our previous work,^21^ where the readers can find the full details. Briefly, the model accounts for ion transport within the electrodes, flow channels, and AEMs. Within the electrode, it incorporates ion partitioning at the electrode–solution interface, ion transport through the cation-exchange polymer (CEP), and ion storage within the carbon micropores. In addition, we now extend this model to incorporate the micropores electrosorption resistance. The flux of ion, *i*, in the CEP, *J*_CEP,*i*_ (mol m^–2^ s^–1^), due to diffusion and electromigration, is governed by the Nernst–Planck equation:^31,32^

$$J_{\mathrm{CEP},i}=-D_{\mathrm{CEP},i}\left(\frac{\partial c_{\mathrm{CEP},i}}{\partial x}+z_{i}c_{\mathrm{CEP},i}\frac{\partial \phi_{\mathrm{CEP}}}{\partial x}\right)$$ 5

where *c*_CEP,*i*_ is the concentration of species *i* in the CEP, *z*_*i*_ is the valence of species *i*, ϕ_CEP_ is the electric potential in the CEP, and *D*_CEP,*i*_ is the effective diffusion coefficient of ions in the CEP, which is related to the diffusion coefficient of ions (*D*_*i*_) in solution according to

$$D_{\mathrm{CEP},i}=\frac{\epsilon f_{\mathrm{CEP}}}{\tau }D_{i}$$ 6

where ϵ is the macroscopic porosity of the porous medium (carbon) and equals the volume fractions of the CEP (*p*_CEP_), τ is macropore tortuosity which is assumed to follow the Bruggeman correlation (i.e., τ = ε^–0.5^), and *f*_CEP_ is a reduction factor that accounts for the lower ion diffusivity in the CEP than in free solution. Across the electrode, we evaluate the ion mass balance according to

$$\frac{\partial }{\partial t}\left(p_{\mathrm{CEP}}c_{\mathrm{CEP},i}+p_{\mathrm{pore}}c_{\mathrm{pore},i}\right)=-\frac{\partial J_{\mathrm{CEP},i}}{\partial x}$$ 7

where *p*_CEP_ and *p*_pore_ are the volume fractions of the CEP and micropores, respectively; and *c*_pore,*i*_ is the concentration of species *i* in the micropores. Additionally, charge neutrality is always maintained for the CEP phase:

$$\sum_{i}z_{i}c_{\mathrm{CEP},i}+X_{\mathrm{CEP}}=0$$ 8

where *X*_CEP_ (mM) is the CEP charge density.

While the previous mathematical model developed for EIP (and in most CDI studies) assume equilibrium partitioning for ions entering or exiting micropores, a recent study suggests the electro-sorption resistance is nontrivial.^33^ Assuming instant equilibrium of the electrical double layers (EDLs) and the associated dynamics, the extended Donnan theory results in local ion depletion zones in the macropores and overestimated the ion transport rate in constant voltage CDI experiment.^33^ The extended Donnan model is modified to capture the resistance for ion entering the micropores from macropores using a formulation resembling the Butler–Volmer equation utilized for describing the charge transfer rate in redox reactions. By accounting for the interfacial resistance arising from the kinetic barrier for ion exchange between the micro- and macropores, the model, which offers a more accurate prediction of the experimental data, has the following form:^33,34^

$$J_{\mathrm{CEP},i}=k[c_{\mathrm{CEP},i}\exp(\alpha_{\mathrm{int}}(-z_{i}(\Delta \phi_{D,p|c}+\Delta \phi_{R,p|c})+\mu_{\mathrm{att}}))-c_{\mathrm{pore},i}\exp((1-\alpha_{\mathrm{int}})(z_{i}(\Delta \phi_{D,p|c}+\Delta \phi_{R,p|c})+\mu_{\mathrm{att}}))]$$ 9

where α_int_ is the interfacial transfer coefficient, Δϕ_D,p|c_ is defined as the dimensionless Donnan potential across the CEP and micropore interface at equilibrium, Δϕ_R,p|c_ is the potential drop across the CEP and carbon micropore interface due to interfacial resistance, and *k* is the transfer rate constant (m s^–1^) and is associated with the accessible ion adsorption sites (*k* = *k*_0_ *C*_st,vol,0_^*n*^, *k*_0_ is the reference transfer rate constant, *C*_st,vol,0_ is the volumetric Stern layer capacitance in the zero-charge limit, and *n* is the exponent term). If the distribution of the ions between the macropores (CEP phase) and micropores (carbon phase) is at equilibrium (i.e., no kinetic barrier for ion transfer), eq 9 is reduced to the interfacial Donnan partitioning equilibrium: *c*_pore,*i*_ = *c*_CEP,*i*_ exp (− *z*_*i*_Δϕ_D,p|c_ + μ_att_) (*J*_CEP,*i*_ = 0 and Δϕ_R,p|c_ = 0). The attraction term, μ_att_, results from interactions between individual ions and the metallic pore surfaces (e.g., image forces).^35^ Instead of being a constant in the modified Donnan model, μ_att_ is a function of the pore ion correlation energy, *E*, and the total ion concentration in the pores, *c*_pore, tot_:

$$\mu_{\mathrm{att}}=\frac{E}{c_{\mathrm{pore},\mathrm{tot}}}$$ 10

In the micropores, the charge density and total ion concentration can be expressed as

$$\sigma_{\mathrm{pore}}=\underset{i}{\sum }z_{i}c_{\mathrm{pore},i}$$ 11

$$c_{\mathrm{pore},\mathrm{tot}}=\underset{i}{\sum }c_{\mathrm{pore},i}$$ 12

The electronic charge density, σ_elec_, can be related to the Stern layer potential drop, Δϕ_s_,

$$\sigma_{\mathrm{elec}}F=C_{\mathrm{st},\mathrm{vol}}\Delta \phi_{s}V_{T}$$ 13

where *F* is the Faraday constant, *C*_st,vol_ is the volumetric Stern layer capacitance (F mL^–1^), and *V*_T_ is the thermal voltage (25.7 mV at 25 °C). The parameter *C*_st,vol_ can be estimated using an empirical expression reported elsewhere:^36,37^

$$C_{\mathrm{st},\mathrm{vol}}=C_{\mathrm{st},\mathrm{vol},0}+{\alpha \sigma }_{\mathrm{pore}}^{2}$$ 14

where *C*_st,vol,0_ is the volumetric Stern layer capacitance in the zero-charge limit estimated by CV tests and α is a coefficient that accounts for the charge dependence of Stern capacitance. In this study, we used typical values for porous carbon materials:^35^ α = 30. The electronic charge is balanced by the ionic charge in the micropores:

$$\sigma_{\mathrm{pore}}+\sigma_{\mathrm{elec}}=0$$ 15

$$\frac{\partial }{\partial t}(p_{\mathrm{pore}}{\overset{-}{\sigma }}_{\mathrm{elec}})=\frac{I}{FL_{\mathrm{elec}}}$$ 16

where ${\overset{-}{\sigma }}_{\mathrm{elec}}$ is the spatial average of electronic charge density, *I* is current density (A m^–2^) and *L*_elec_ is the electrode thickness. Additional boundary conditions are specified in S1 in Supporting Information. Ion transport in the spacer channels is described in S2 in Supporting Information.

Based on this model, the potential drop between the midplane of the flow channel (where the reference electrodes are located) and the current collector (Figure 1d) is

$$V_{\mathrm{elec}}=(\phi_{\mathrm{CEP}}+\Delta \phi_{D,p|c}+\Delta \phi_{R,p|c}+\Delta \phi_{s}+\Delta \phi_{\mathrm{sp},\mathrm{half}})\frac{RT}{F}+IR_{\mathrm{ex}}$$ 17

where Δϕ_sp,half_ is the potential drop from the midplane of the spacer channel to the CSE interface due to ionic resistance, and *R*_ex_ is the electronic resistance in the percolated carbon phase and is simulated using the following empirical equation:^38,39^

$$R_{\mathrm{ex}}=\frac{1}{\sigma_{0}(p_{c}-p_{c,0}{)}^{t}}$$ 18

where σ_0_ is a pre-exponential factor that depends on the conductivity of the material, *p*_*c*_ is the volume fraction of carbon in the composite (*p*_c_ + *p*_CEP_ = 1), *p*_c,0_ is the percolation threshold of the carbon phase and *t* is the conductivity exponent. The volume fraction of AC (*p*_AC_) can be calculated as the volume ratio of AC in the entire CSE:

$$p_{\mathrm{AC}}=\frac{\upsilon_{\mathrm{AC}}}{\upsilon_{\mathrm{tot}}}=\frac{\frac{w_{\mathrm{AC}}}{\rho_{\mathrm{AC}}}}{\frac{w_{\mathrm{AC}}}{\rho_{\mathrm{AC}}}+\frac{w_{\mathrm{CB}}}{\rho_{\mathrm{CB}}}+\frac{w_{\mathrm{CEP}}}{\rho_{\mathrm{CEP}}}}$$ 19

where υ_AC_ and υ_tot_ are the volume of AC and total volume of carbon and polymer, respectively, *w*_AC_, *w*_CB_, and *w*_CEP_ are the mass fraction of AC, CB, and CEP, respectively, and ρ_AC_, ρ_CB_, and ρ_CEP_ are the density of AC, CB, and CEP, respectively. The volume fraction of CB and CEP can be calculated similarly. The ion flux, current efficiency, and SEC_*i*_ are calculated using eqs 1–3. The resistance in spacer channel (*R*_sp_, Ohm m^2^) is calculated by

$$R_{\mathrm{sp}}=\frac{\int_{0}^{t_{\mathrm{hc}}}\Delta \phi_{\mathrm{sp},\mathrm{half}}dt+\int_{t_{\mathrm{hc}}}^{t_{c}}\Delta \phi_{\mathrm{sp},\mathrm{half}}dt}{it_{c}}$$ 20

Similarly, the resistance from electrosorption (*R*_ES_, Ohm m^2^) and from IEP (*R*_IEP_, Ohm m^2^) can be calculated by

$$R_{\mathrm{ES}}=\frac{\int_{0}^{t_{\mathrm{hc}}}\Delta \phi_{R,p/c}dt+\int_{t_{\mathrm{hc}}}^{t_{c}}\Delta \phi_{R,p/c}dt}{it_{c}}$$ 21

$$R_{\mathrm{IEP}}=\left[\int_{0}^{t_{\mathrm{hc}}}(\phi_{\mathrm{CEP}}+\Delta \phi_{D,p|c}+\Delta \phi_{s})dt+\int_{t_{\mathrm{hc}}}^{t_{c}}(\phi_{\mathrm{CEP}}+\Delta \phi_{D,p|c}+\Delta \phi_{s})dt\right]/\left[it_{c}\right]$$ 22

The electrode parameters and system dimensions used for the simulation are summarized in Table S1 in Supporting Information.

## Results and Discussion

### Trade-Off between Ionic and Electronic Resistances with Different Carbon Fractions

To understand how electrode composition influences EIP performance, we first investigated the effect of the carbon fraction using both AC and CB electrodes. Because AC provides high ion storage capacity while CB offers superior electronic conductivity, AC- and CB-based electrodes exhibit distinct electron percolation behavior and ionic electro-sorption accessibility, leading to different optimal carbon-to-polymer ratios. Overall, the balance between carbon and polymer fractions reveals a fundamental trade-off between ionic resistance in the polymer phase and electronic resistance in the carbon phase, resulting in distinct performance optima (Figure 2). Although spacer channel and interfacial resistances were also considered, they showed negligible variation with different carbon fractions, and the observed trends are primarily explained by the balance between ionic and electronic resistances. We note that the absolute magnitude of the spacer-channel resistance in our setup is elevated because we used a relatively thick spacer to accommodate the reference electrode for in situ potential measurements. This design choice was made for diagnostic purposes and is not representative of a practical EIP module. In an application-oriented cell, the spacer/channel thickness can be substantially reduced, which would proportionally lower the spacer channel ohmic loss and shift the overall resistance toward electrode contributions.

**Figure 2:**
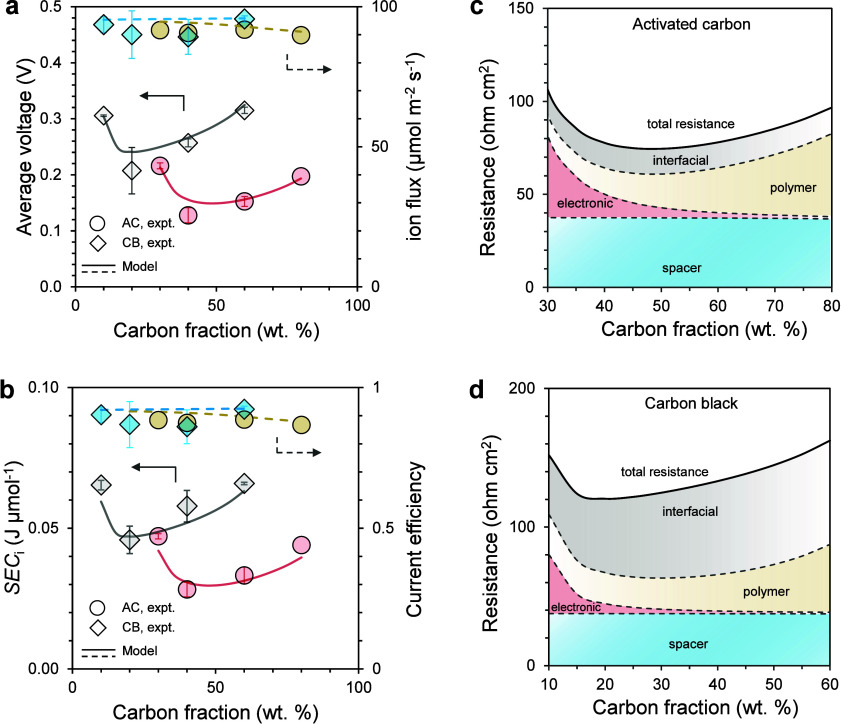
(a, b) Average cell voltage (left axis) and ion flux (right axis) (a) and specific energy consumption (SEC_*i*_, left axis) and current efficiency (right axis) (b) as a function of carbon fraction for electrodes prepared with activated carbon (AC, red circles) or carbon black (CB, blue diamonds). Experimental data (scatters) are compared with model predictions (lines). (c, d) Decomposition of electrode resistance into contributions from spacer, electronic, polymeric, and interfacial components for AC-based (c) and CB-based (d) electrodes. The half-cycle time is 5 s and current density is 20 A m^–2^.

For AC-based electrodes, the cell voltage was very high at low carbon fraction and relatively insensitive to carbon fraction beyond 30% (Figure 2a, minimum at 40%). The ion flux and current efficiency remained nearly constant across the tested compositions (Figure 2a,b). Under constant-current operation, the ion-specific energy consumption (SEC_*i*_) followed the trend of voltage, reaching an optimum of 28 mJ μmol^–1^ at a carbon fraction of 40% (Figure 2b). Model simulations are in good agreement with the experimental results. Resistance analysis showed that spacer channel and the polymer-micropore interfacial resistance contributions were largely unaffected by the carbon fraction. With increasing carbon fraction (i.e., reducing the polymer fraction), electronic resistance decreased due to better percolation, but noninterfacial ionic resistance increased due to less transport pathways and larger tortuosity with less volume filled with CEP (Figure 2c).

Electrodes made of CB and CEP, despite their lower ion storage capacity, also exhibited the characteristic convex dependence of voltage on carbon fraction but with different magnitudes (Figure 2a). The optimal SEC_*i*_ (0.046 J μmol^–1^) occurred at a 20:80 CB-to-polymer ratio and was much higher than for AC electrodes (Figure 2b). The interfacial resistance of CB electrodes is consistently larger than AC electrodes, which is attributed to fewer accessible electrosorption sites of CB. However, their electronic resistance was much lower than that of AC electrodes (Figure 2d). Notably, AC electrodes lost percolation below ∼20% carbon, leading to a sharp increase in electronic resistance and nearly an order-of-magnitude rise in SEC_*i*_ relative to the optimum (Figure S2 in Supporting Information). By contrast, CB electrodes retained percolation down to 10% carbon, with electronic resistance increasing 2-fold and SEC_*i*_ rising by only ∼50%. On the other hand, the polymer not only provided ion transport pathways but also served as a binder for carbon particles. Electrodes with less than 20% polymer exhibited poor mechanical integrity and tended to disintegrate. These observations indicate that both carbon and polymer contents have lower bounds set by electronic percolation and mechanical stability, respectively.

### Combining AC and CB: Balancing Capacity and Conductivity

To combine the high electronic conductivity of CB with the high ion storage capacity of AC, we next investigated the influence of AC/CB ratios on EIP electrode performance. The polymer phase was fixed at 60% and the carbon phase at 40%, while the AC fraction in the carbon phase was adjusted from 0 to 100%. In this system, electrode performance reflects the interplay between ion storage capacity and electrical conductivity.

Cyclic voltammetry (CV) showed a nearly linear increase in electrode capacitance with AC fraction. Pure CB exhibited a very low capacitance of ∼2.5 F g^–1^, while pure AC reached ∼138 F g^–1^ (Figures 3a and S3). Electrochemical impedance spectroscopy (EIS) further revealed very different ionic and electronic transport characteristics between CB and AC: CB-based electrodes displayed higher electronic conductivity but also greater internal ion-transport resistance within the composite electrode microstructure compared to AC-based electrodes (Figures 3b and S4). The high-frequency real-axis intercept corresponds to the total ohmic resistance from the electrolyte solution, electronic resistance of current collectors and contact resistances. This resistance remained nearly constant (∼3.2–3.5 Ω) across all compositions. A small semicircle appears in the midfrequency region and decreases markedly with increasing CB content, becoming nearly indistinguishable for 100% CB (Figure 3b). Because these carbon-based electrodes operate predominantly via non-Faradaic EDLs charging, this semicircle is not attributed to Faradaic charge-transfer kinetics. Instead, it reflects interfacial and distributed polarization processes within the porous composite electrode, including finite electronic percolation between carbon particles and ionic transport through the tortuous polymer network. At low frequencies, the impedance increases with CB fraction (Figure S3), consistent with enhanced internal ion-transport resistance within the composite microstructure due to reduced accessibility of electro-sorption sites.

**Figure 3:**
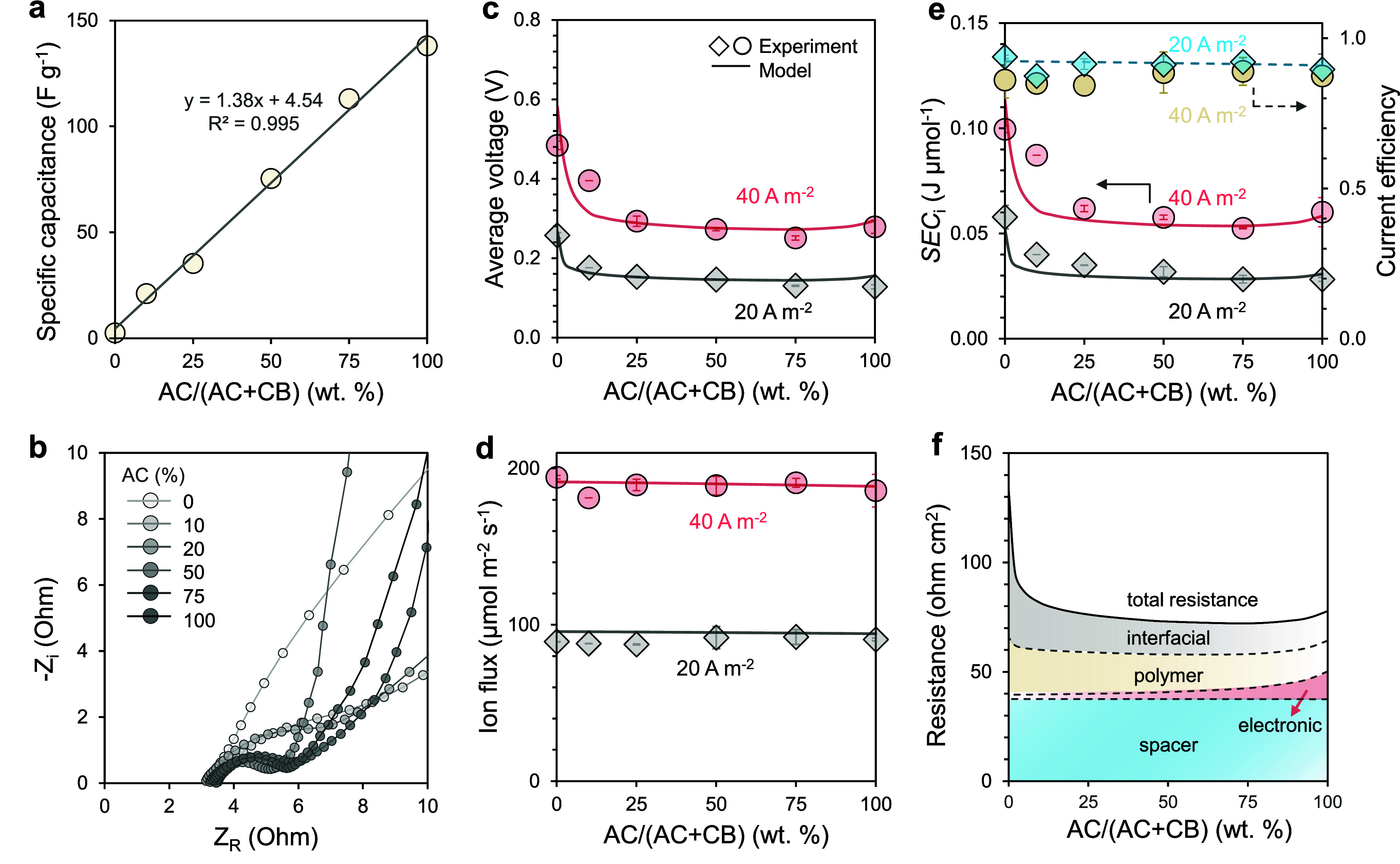
(a) Specific capacitance of composite electrodes as a function of AC in carbon fraction from cyclic voltammetry test, showing a near-linear increase with AC content. (b) Nyquist plots from electrochemical impedance spectroscopy (EIS) of electrodes with different AC fractions in carbon. The figure shows high-frequency regions. (c) Average cell voltage, (d) ion flux, and (e) specific energy consumption (SEC_*i*_, left axis) and current efficiency (right axis) as a function of AC fraction at two current densities (20 and 40 A m^–2^). (f) Modeled decomposition of resistance into spacer, electronic, polymeric, and interfacial contributions as a function of AC fraction. The polymer weight fraction in the electrodes is 60% and the AC and CB content in the carbon phase are varied systematically. The half-cycle time is 5 s.

We then evaluated desalination performance with a 5-s cycle time at various current densities. As the AC fraction decreased from 100 to 25% of the carbon phase, the electrode capacity was reduced accordingly by three-quarters, but desalination performance was only modestly affected (Figure 3c–e). The voltage drop and SEC_*i*_ only increased slightly, and the ion flux and current efficiency remained stable. These results indicate that, under constant-current operation with fast circuit-switching, electrode capacitance does not directly limit desalination performance once it exceeds a minimum threshold. However, when the AC fraction decreased further from 25 to 0% (i.e., the carbon phase comprises only CB), voltage drop and SEC_*i*_ rose sharply. While electrodes with only CB in carbon phase have negligible capacitance, they could still sustain continuous desalination, confirming that achieving desalination in EIP is not strictly constrained by electrode capacitance. However, this operation occurred at the expense of substantially higher energy consumption. Thus, while increasing CB content lowers electronic resistance, the severe reduction in available electro-sorption sites below ∼25% AC content significantly elevates the energy consumption, setting a practical lower limit for AC content.

Unlike conventional CDI whose performance typically benefits from a higher electrode capacity, the performance of EIP is relatively insensitive to electrode capacity once it exceeds a certain threshold owing to its fast-switching mechanism. In the system tested, the interfacial resistance was too large when the carbon phase contained only CB, but became reasonably small once the AC content exceeds ∼25% (Figure 3f). Varying the AC content beyond 25% affects both the interfacial and electronic resistances in opposite directions, but neither to an extent that can exert sizable impact on the overall resistance.

### Switching Frequency Affects Potential Window but Not Energy Consumption

Circuit switching in EIP allows rapid transitions between charging and discharge half-cycles without solution mixing. We hypothesize that operating EIP with short half-cycles, i.e., charging and discharge with high frequencies, can potentially reduce the SEC_*i*_ of EIP. The rationale behind such a hypothesis is that charging the electrodes to a greater extent (i.e., a higher saturation level) intrinsically requires a higher voltage, which translates to more energy consumed per ion removed.

To test this hypothesis, we investigated the impact of half-cycle time on EIP performance under constant-current operation. Specifically, we compare two electrodes with very different capacitances, including a low-capacity electrode (0% AC, 100% CB) and a high-capacity electrode (75% AC, 25% CB) (Figure 3a). In both cases, charging and discharge with a longer half cycle time results in a larger shift in electrode potential due to increased saturation level (Figure 4a,b), partially consistent with our hypothesis. With the low-capacity electrode, the change in electrode potential with the same amount of salt removed is substantially larger than that with the high-capacity electrode. This observation is also consistent with our hypothesis, as an electrode with a higher capacity was relatively less saturated than that with a lower capacity with the same amount of salt adsorption, which consequently induces less shift in electrode potential.

**Figure 4:**
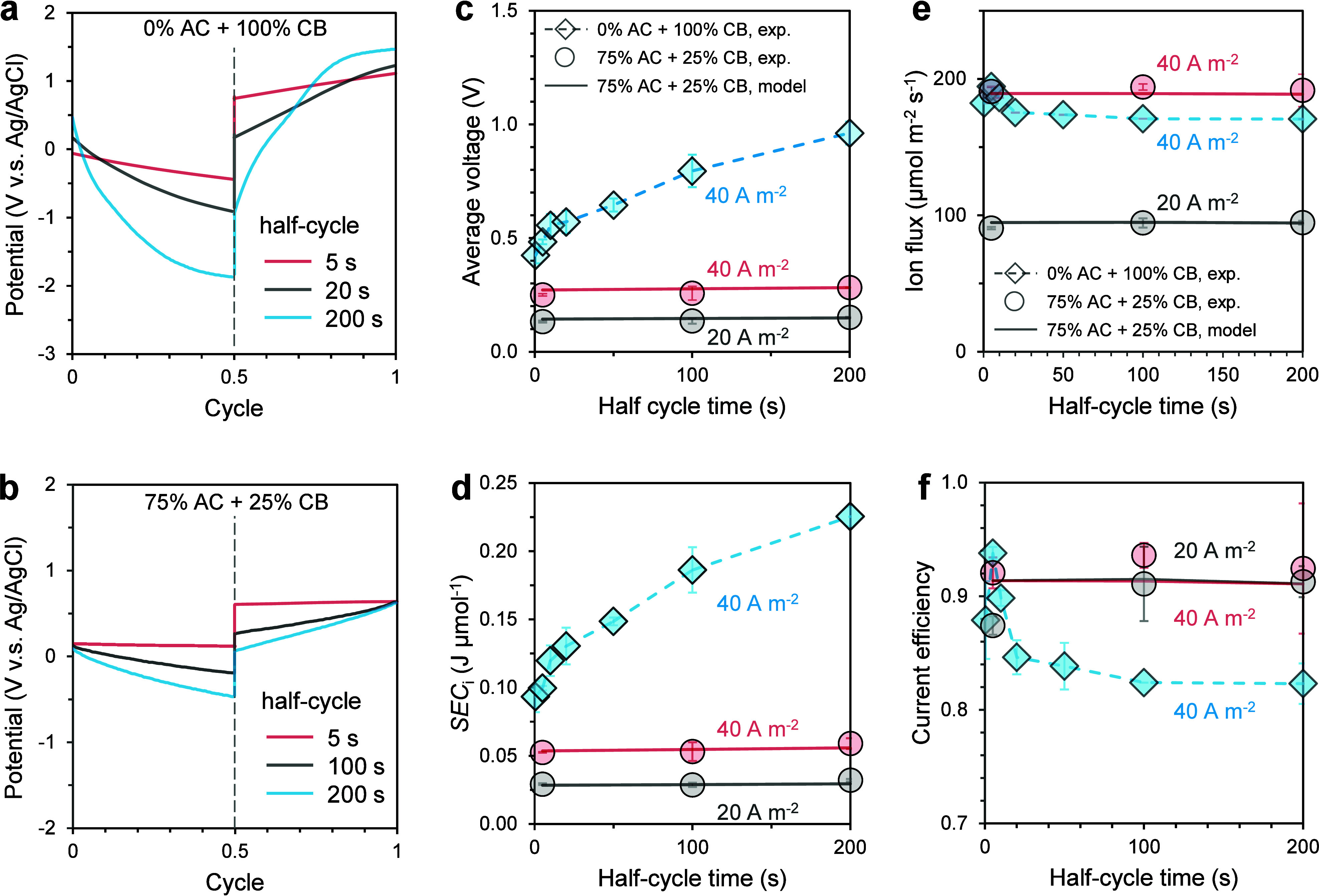
(a,b) Electrode potential profiles during charging/discharge cycles at different half-cycle times for electrodes with 0% AC + 100% CB (a) and 75% AC + 25% CB (b). (c) Average cell voltage, (d) SEC_*i*_ (e) ion flux, and (f) current efficiency as a function of half-cycle time with 75% AC + 25% CB (circles) and 0% AC + 100% CB (diamonds). Experimental data (symbols) are compared with model predictions (lines). Electrolysis begins to occur at longer half-cycle times with 0% AC + 100% CB. Panels b–d share the same color and marker codes. The average voltage in panel (c) is obtained by time-averaging the instantaneous charging and discharging potentials shown in panels (a) and (b) over multiple cycles.

With the low-capacity electrode, the average voltage and SEC_*i*_ of the EIP process increased systematically with a longer charging half cycle (Figure 4c,d). Such changes were attributable to the high and increasing saturation level of the low-capacity electrode when the charging half cycle time increased. At first glance, this observation appears to support our hypothesis that charging the electrode to a higher saturation level consumes more energy. However, despite the appreciable changes in electrode potential over longer charging half cycles for the high-capacity electrode (e.g., 200 s in Figure 4b), very little change was observed in the average voltage and SEC_*i*_ (Figure 4c,d). Therefore, the substantial increase in average voltage and SEC_*i*_ with the low-capacity electrode is not the consequence of a higher saturation level in a purely capacitive charging process, but a result of partial electrolysis as the CB particles become increasingly saturated. Such an explanation is supported by the lower ion flux (Figure 4e) and current efficiency (Figure 4f) for the EIP process with the low-capacity electrodes as the half-cycle time increased. In addition, we measured the pH of the diluate and concentrate reservoirs at the end of EIP operation. For CB-based electrodes operated with long half-cycle times (200 s), the pH of the diluate and concentrate streams shifted from 6.8 to approximately 6.3 and 4.7, respectively, indicating the occurrence of parasitic Faradaic reactions, most notably oxygen evolution during discharge. In contrast, under short half-cycle operation, the pH in both streams remained close to neutral. Consistent with these observations, no visible gas evolution was detected during short half-cycle operation, whereas gas bubbles were observed during prolonged half-cycle operation. These results confirm that partial electrolysis contributes to the elevated voltage and energy consumption observed at long half-cycle times for low-capacity electrodes.

Why does increasing electrode potential not lead to more energy consumption as we hypothesized, as long as no electrolysis reaction occurs as in EIP with high-capacity electrodes? To elucidate the underlying mechanism, we analyzed the temporal evolution of CSE potential versus Ag/AgCl reference electrode and cumulative SEC_*i*_ across different components of the EIP system with a high-capacity electrode (75% AC, 25% CB). The total voltage drop (colored regions in Figure 5a,b) includes contributions from the Donnan and Stern potentials (EDL potential drop), ionic resistance within the polymer, interfacial resistance for electrosorption, ionic resistance in the spacer, and electronic resistance within the electrode. Potential profiles were compared for short (5 s) and long (200 s) half-cycle times (Figure 5a,b).

**Figure 5:**
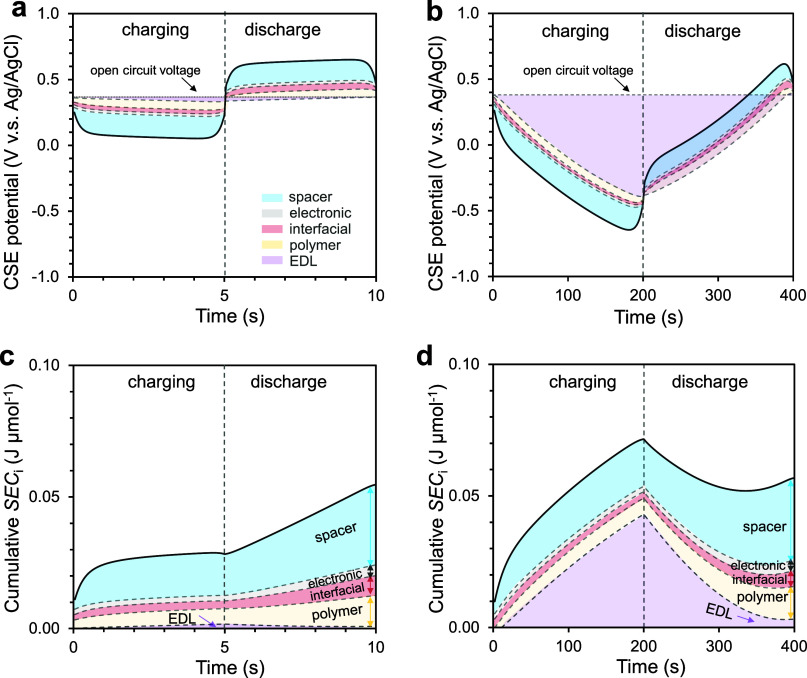
(a, b) Simulated voltage contributions from different components to the CSE potential profiles versus Ag/AgCl reference electrodes in EIP system under short (5 s, a) and long (200 s, b) half-cycle durations. Temporal voltage decomposed into contributions from electronic, spacer, interfacial, polymeric, and EDL resistances during charging and discharge. Short half-cycles yield narrow potential windows with negligible EDL contribution, whereas long half-cycles expand the potential window and introduce significant EDL energy storage during charging. Both charging and discharge half-cycles are driven by an externally applied current and consume electrical energy; the reduced voltage during discharge reflects reuse of electrostatic energy stored in the CSE during charging assuming energy recovery. (c, d) Cumulative specific energy consumption (SEC_*i*_) under short (5 s, c) and long (200 s, d) half-cycle durations. The energy storage in the EDLs during charging and energy reuse during discharge results in relatively stable overall energy consumption across switching frequencies. The simulated current density is 40 A m^–2^. The electrode is the optimized 75% AC + 25% CB electrode.

At a constant current density, a shorter half cycle resulted in smaller changes in charging and discharge voltages, whereas a longer half cycle led to larger charging and discharge voltage variations. The larger potential window with a longer half cycle primarily arose from the greater changes in EDL potential, while contributions from interfacial, spacer, and electronic resistances remained relatively constant regardless of the half cycle time (Figure 5a,b). The energy stored in the carbon-phase EDLs was negligible in a 5 s charging half cycle but became sizable when the charging half cycle time increased to 200 s (Figure 5c,d). Therefore, a longer half cycle increased the energy input during charging, which is consistent with our hypothesis. However, the additional energy input stored in the EDL during the charging half cycle is subsequently released and reused to drive the ion desorption in the discharge half cycle. As a result, the cell voltage during the discharge half-cycle is reduced relative to that during the charging half-cycle. At the beginning of discharge, the negative potential drop from CSE to reference electrode leads to the decreasing cumulative SEC_*i*_, which indicates electrical energy recovery (negative power), primarily from relaxation of the built electrical energy in EDL during charging. However, EDL formation and dissipation have no collective contribution to the cumulative SEC_*i*_ over a full charging-discharge cycle. In other words, our hypothesis regarding the impact of half cycle time on energy consumption is only partially correct when applied to the charging half cycle, but not to a full cycle. The SEC_*i*_ of an EIP process is relatively independent of the half cycle time as long as it is not too long to fully saturate the electrode and introduce electrolysis.

## Implications

Supported by both experiments and model simulations, this study highlights several electrode design and operational principles critical to optimizing the performance of EIP for desalination. The overall principle for electrode composition optimization is to balance the resistances between ionic and electronic transport. While the ion exchange polymer provides the pathways and reduces the resistance for ion transport, the carbon phase imparts electronic conductivity, and in the case of AC, ample ion storage capacity. Too much polymer restricts electron transfer, while too much carbon hinders ion mobility. Therefore, identifying the optimal balance is central to maximizing desalination efficiency.

While electrode capacity is widely regarded as a limiting factor in conventional CDI, our results show that desalination performance in EIP is relatively insensitive to capacity. Only when capacity is too small (as in the case of 100% CB) and the half cycle time is relatively long did we observe the deterioration of desalination performance due to the presence of electrolysis. In the absence of electrolysis, while charging with a longer half cycle time increases the electrode potential and consumes more energy to form an EDL with more stored charges, it does not affect the overall energy consumption for a full charging-discharge cycle due to automatic utilization of the energy stored in the EDL in driving the ion desorption process. This independence of desalination performance on half cycle time renders careful optimization of EIP operation unnecessary for desalination, which reduces the degree of freedom for optimizing EIP performance. Therefore, future efforts in improving EIP performance should focus on the design of electrodes and cell stacks.

## Supplementary Material



## Notes

S1: boundary conditions applied in the theoretical simulation of the EIP process; S2: ion transport in the spacer channels; Table S1: electrode parameters and system dimensions used in simulation; Figure S1: schematic of the experimental configuration used for electrochemical ion pumping (EIP) experiments; Figure S2: low energy efficiency and high electronic resistance for the electrode with 20% AC and 80% polymer; Figure S3: cyclic voltammetry of electrodes with varying AC fractions in carbon; and Figure S4: full-range Nyquist plots from electrochemical impedance spectroscopy (EIS) of electrodes with different AC fractions in carbon.(PDF)
