# Supplementary material for: Principles for Optimal Electrode Design and Operation for Desalination with Electrochemical Ion Pumping
Source: Environ Sci Technol. 2026 Feb 23;60(9):7544–54. doi: 10.1021/acs.est.5c15799 (PMC12980839; doi:10.1021/acs.est.5c15799)
Supplement: Supplementary file 1 [file es5c15799_si_001.pdf]

Supplementary information

Principles for Optimal Electrode Design and Operation for Desalination with Electrochemical Ion Pumping

Manuscript Submitted to

Environmental Science & Technology

October 2025

Weifan Liu[a, c], Longqian Xu[a, c *], Shihong Lin[a, b, c *]

[a] Department of Civil and Environmental Engineering, Vanderbilt University, Nashville, Tennessee 37235-1831, USA

[b] Department of Chemical and Biomolecular Engineering, Vanderbilt University, Nashville, Tennessee 37235-1831, USA

[c] Department of Civil and Environmental Engineering, Rice University, Houston, Texas 77005, United States

Contents:

7 Pages

2 Supporting Texts

1 Supporting Table

4 Supporting Figures

S1. Boundary conditions applied in the theoretical simulation of the EIP process. The boundary conditions were adopted from a recent theoretical study on EIP.[1] Specifically, with CEP charge density (X) and the concentration in the bulk solution (without considering concentration polarization in this work), the electric potential of the CEP at the CEP/solution interface, can be evaluated using the following equation:

$$X = 2c_{\infty,\mathrm{dil}}\sinh\left(\phi_{\mathrm{CEP/dil}} + \phi_{\infty,\,\mathrm{dil}}\right) \tag{S1a}$$

$$X = 2c_{\infty,\mathrm{conc}}\sinh\left(\phi_{\mathrm{CEP/conc}} + \phi_{\infty,\,\mathrm{conc}}\right) \tag{S1b}$$

where $c_{\infty,\mathrm{dil}}$ and $c_{\infty,\mathrm{conc}}$ are diluate and concentrate bulk concentrations, respectively; $\phi_{\mathrm{CEP/dil}}$ and $\phi_{\mathrm{CEP/conc}}$ are the electric potential of the CEP next to the diluate/CEP interface and the CEP/concentrate interface, respectively. When the electrode is in the adsorption step, $\phi_{\infty,\,\mathrm{dil}}$ is set to zero; when the electrode is in the desorption step, $\phi_{\infty,\,\mathrm{conc}}$ is set to zero.

To solve the coupled ion transport and partitioning, we need boundary conditions corresponding to the operation of EIP. The most important boundary condition is the zero current at the CEP/solution interface for the solution in the disconnected (open) circuit. The zero current boundary condition can be written as

$$\frac{I}{F} = \sum_i z_i J_{\mathrm{CEP},i,\,\mathrm{open}} = 0 \tag{S2}$$

where $J_{\mathrm{CEP},i,\,\mathrm{open}}$ is the flux of species i across the interface in the open circuit.

At each CEP–solution interface, the partitioning equilibria between the concentrations in the bulk solution and CEP phase concentration are described by Donnan equilibrium:

$$c_{\mathrm{CEP/dil},\,i} = c_{\infty,\mathrm{dil}}\exp\left[-z_i\left(\phi_{\mathrm{CEP/dil}} - \phi_{\infty,\,\mathrm{dil}}\right)\right] \tag{S3a}$$

$$c_{\mathrm{CEP/conc},\,i} = c_{\infty,\mathrm{conc}}\exp\left[-z_i\left(\phi_{\mathrm{CEP/conc}} - \phi_{\infty,\,\mathrm{conc}}\right)\right] \tag{S3b}$$

where $c_{\mathrm{CEP/dil},\,i}$ and $c_{\mathrm{CEP/conc},\,i}$ are the CEP phase concentrations of species i near the diluate and concentrate interfaces, respectively.

S2. Ion transport in the spacer channels. In the spacer channels, we assume plug flow and neglect axial dispersion and electromigration in the flow direction. Concentration profiles develop in the direction perpendicular to the flow direction. The ion transport in the x-direction in spacer channel can be describe by the Nernst-Planck equation:

$$J_{\mathrm{sp},\,i} = -\,D_i\left(\frac{\partial c_{\mathrm{sp},\,i}}{\partial x} + z_i c_{\mathrm{sp},\,i}\frac{\partial \phi_{\mathrm{sp}}}{\partial x}\right) \tag{S4}$$

where $c_{\mathrm{sp},\,i}$ is the concentration of species i in the spacer channel, and ϕ_{sp} is the electric potential in the spacer channel. The overall salt balance in the spacer channel can be described by the following partial differential equation:

$$\frac{\partial c_{\mathrm{sp},\,i}}{\partial t} = -\frac{\partial J_{\mathrm{sp},i}}{\partial x} + \frac{c_{inf} - c_{\mathrm{sp},\,i}}{\tau_{sp}} \tag{S5}$$

where $c_{\mathrm{sp},\,i}$ is the concentration of ion i in the spacer, c_{inf} is the salt concentration of the influent to the spacer channel, and τ_{sp} is the hydraulic retention time ($\tau_{sp} = A_{CSE}L_{sp}/Q$, where A_{CSE} is the surface area of an electrode, L_{sp} is the thickness of spacer channel, Q is the volumetric flow rate). In a semi-batch mode with recirculation, c_{inf} is the same as the concentration in the diluate or concentrate tank and can be expressed as:

$$\frac{\partial c_{inf}}{\partial t} = \frac{c_{sp} - c_{inf}}{\tau_{tank}} \tag{S6}$$

where τ_{tank} is the hydraulic retention time in the diluate or concentration tank ($\tau_{tank} = V_{tank}/Q$, where V_{tank} is the solution volume in the feed or concentrate tank and Q is the volumetric flow rate). In the flow channels, the condition of electroneutrality is satisfied everywhere:

$$\sum_i z_i c_{\mathrm{sp},\,i} = 0 \tag{S7}$$

The parameters used in the theoretical calculation and experimental setup are specified in Table S1.

Table S1. Electrode parameters and system dimensions used in simulation.

symbols	description	value	unit	ref.
D_{Na^+}	Na^+ diffusion coefficient	1.33×10^{-9}	$m^2\ s^{-1}$	
D_{Cl^-}	Cl^- diffusion coefficient	2.03×10^{-9}	$m^2\ s^{-1}$	
f_{CEP}	diffusion reduction factor	0.02	unit	[2]
f_{AEM}	diffusion reduction factor	0.02	unit	[3]
X	polyelectrolyte charge density	-2.5	M	[4]
X_{AEM}	AEM charge density	2.5	M	[4]
E	ion correlation energy	300	$kT\ mol\ m^{-3}$	[5]
α	factor for charge dependence of Stern capacitance	30	unit	[5]
$C_{\mathrm{st,vol,0,AC}}$	zero-charge capacitance of AC	138	$F\ mL^{-1}$	
$C_{\mathrm{st,vol,0,CB}}$	zero-charge capacitance of CB	2.5	$F\ mL^{-1}$	
A_{CSE}	surface area of an electrode	6	cm^2	
τ_{sp}	hydraulic retention time in spacer channel	5.8	s	
τ_{tank}	hydraulic retention time in the diluate or concentration tank	30	s	
k_0	ion transfer rate constant	5×10^{-7}	$m\ s^{-1}$	
n	exponent for ion transfer	0.5	unit	
α_{int}	interfacial transfer coefficient	0.05	unit	
ρ_{AC}	density of AC	1	$g\ mL^{-1}$	
ρ_{CB}	density of CB	0.75	$g\ mL^{-1}$	
ρ_{CEP}	density of CEP	1.2	$g\ mL^{-1}$	
$p_{\mathrm{AC},\,0}$	percolation threshold of AC	0.14	unit	
t_{AC}	conductivity exponent of AC	3	unit	
$p_{\mathrm{CB},\,0}$	percolation threshold of CB	0.06	unit	
t_{CB}	conductivity exponent of CB	2	unit	
σ_0	resistance pre-exponential factor	2.67	$ohm\ cm^{-2}$	

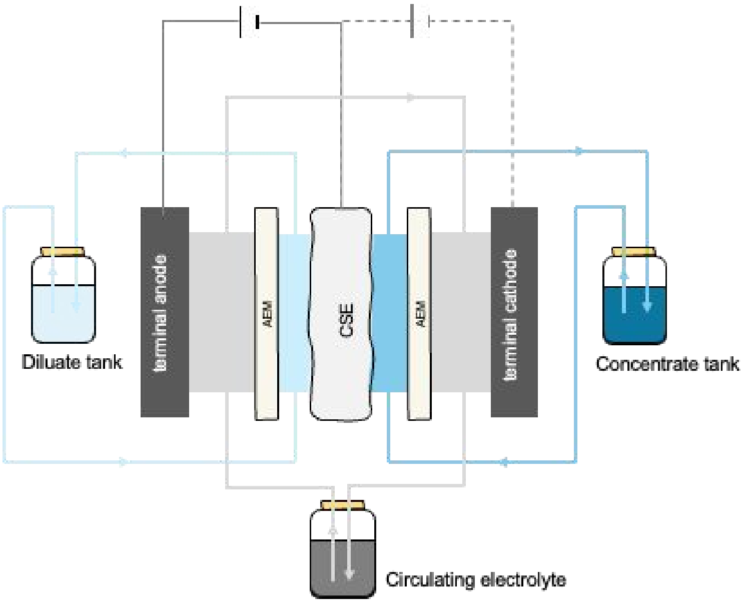

Figure S1. Schematic of the experimental configuration used for electrochemical ion pumping (EIP) experiments. The system consists of an EIP cell connected to separate diluate and concentrate reservoirs via continuous flow loops. Solution is circulated through the diluate and concentrate channels on either side of the cation-shuttling electrode (CSE) using peristaltic pumps, while relay-controlled circuit switching alternately connects the CSE to the terminal anode and cathode.

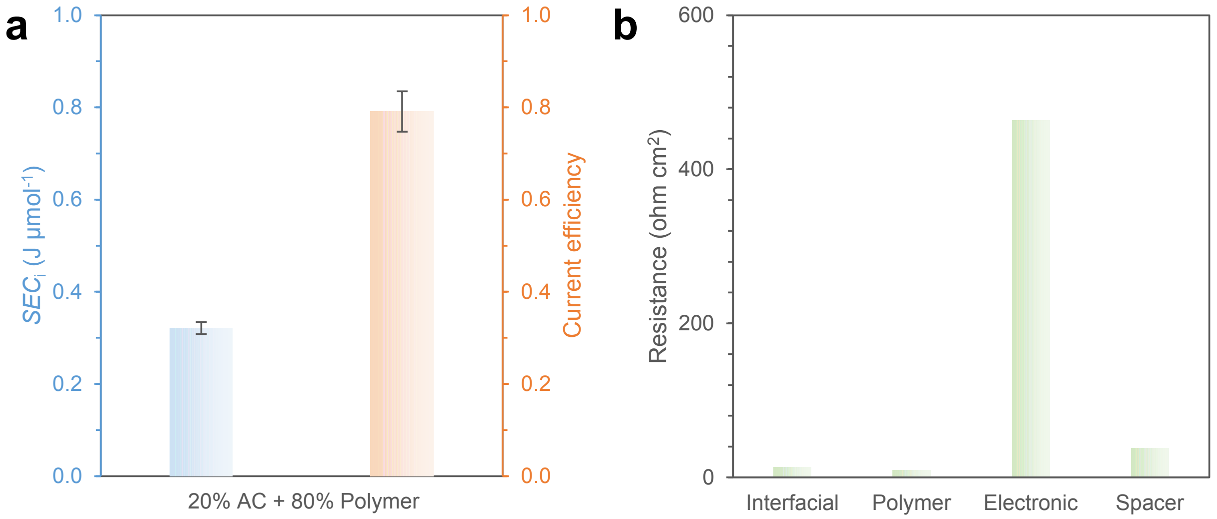

Figure S2. Low energy efficiency and high electronic resistance for the electrode with 20% AC and 80% polymer. (a) specific energy consumption (SECi, left axis) and current

efficiency (right axis) at 20% AC and 80% polymer. (b) Simulated contributions of different resistances.

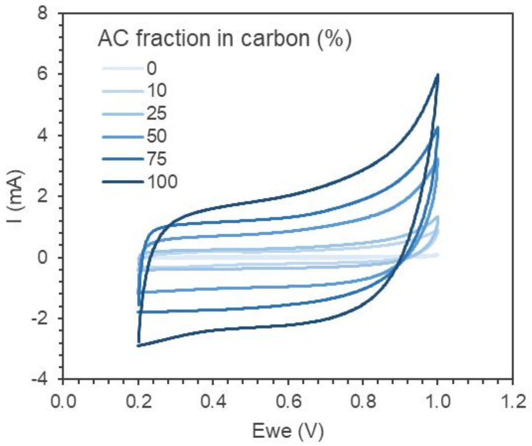

Figure S3. Cyclic voltammetry of electrodes with varying AC fractions in carbon.

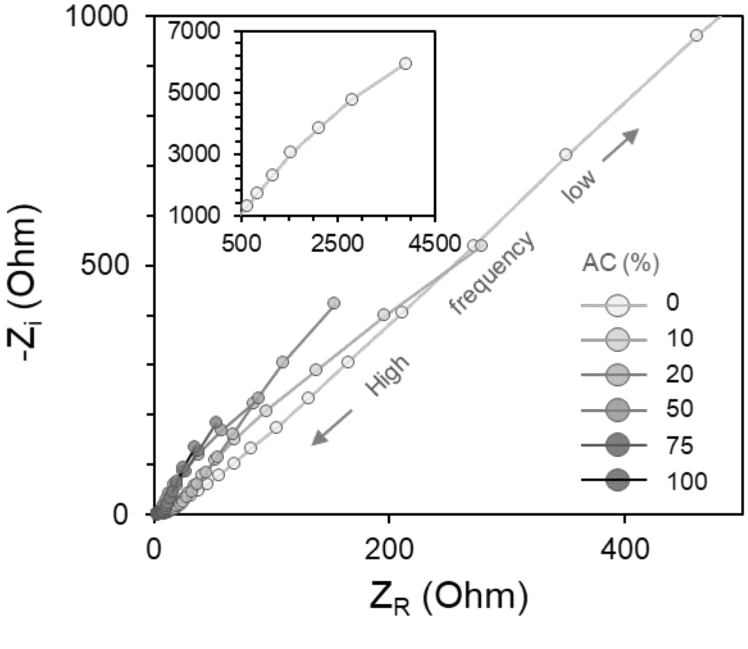

Figure S4. Full-range Nyquist plots from electrochemical impedance spectroscopy (EIS) of electrodes with different AC fractions in carbon. Inset shows low-frequency regions.

References

(1) Liu, W.; Dykstra, J. E.; Biesheuvel, P. M.; Xu, L.; Lin, S. Theory for Dynamic Ion Transport in Ion-Shuttling Electrodes for Electrochemical Ion Pumping. *Nature Water* **2025**, *3* (9), 1025–1037. https://doi.org/10.1038/s44221-025-00480-1.

(2) Mubita, T. M.; Porada, S.; Biesheuvel, P. M.; van der Wal, A.; Dykstra, J. E. Strategies to Increase Ion Selectivity in Electrodialysis. *Sep Purif Technol* **2022**, *292*, 120944. https://doi.org/10.1016/J.SEPPUR.2022.120944.

(3) Dykstra, J. E.; Zhao, R.; Biesheuvel, P. M.; Van der Wal, A. Resistance Identification and Rational Process Design in Capacitive Deionization. *Water Res* **2016**, *88*, 358–370. https://doi.org/10.1016/J.WATRES.2015.10.006.

(4) Wang, L.; Liang, Y.; Zhang, L. Enhancing Performance of Capacitive Deionization with Polyelectrolyte-Infiltrated Electrodes: Theory and Experimental Validation. *Environ Sci Technol* **2020**, *54* (9). https://doi.org/10.1021/acs.est.9b07692.

(5) Biesheuvel, P. M.; Porada, S.; Levi, M.; Bazant, M. Z. Attractive Forces in Microporous Carbon Electrodes for Capacitive Deionization. *Journal of Solid State Electrochemistry* **2014**, *18* (5). https://doi.org/10.1007/s10008-014-2383-5.
